# New Substitute for Rubber

**Published:** 1880-12

**Authors:** 


### NEW SUBSTITUTE FOR RUBBER.

This artificial composition, which answers the purpose of genuine caoutchouc or gutta percha, can be employed, according to Dankworth and Sanders, of St. Petersburg, either alone or in connection with other resinous substances. According to *Ackerman's Gewerbezeitung*, this new product affords an inexpensive means for a perfect isolation of wires for electrical purposes. The composition is elastic, tough, not so sensitive to external influences as caoutchouc or gutta percha, and is not injured by high pressure or high temperature. It is prepared in the following manner:

A quantity of coal tar oil, or equal parts of coal and wood tar oil, which is to constitute a third part of the whole mixture, is poured into a large kettle, together with an equal quantity of hemp oil, and is heated for several hours, either over steam or an open fire, to a temperature between 252° and 288° Farenheit (it should not exceed the latter), until the masss becomes so ductile that it can be drawn in long threads, and the remaining third, consisting of a quantity of linseed oil, which has been thickened by boiling, is then added.

With this composition from five to ten per cent of ozokerite and some spermaceti should be mixed. The mass is then heated again for some hours at the same temperature as above, and finally from seven to twelve per cent of sulphur is added. The mixture thus obtained is cast into forms and treated the same as caoutchouc.

The proportions of the three oils may be slightly varied according to the practical purposes for which the composition is to be used.

